# Use of Long-Acting Injectables in Borderline Personality Disorder: What Do We Know?

**DOI:** 10.1192/j.eurpsy.2024.281

**Published:** 2024-08-27

**Authors:** E. D. S. Almeida, J. Abreu, R. P. Vaz, J. Martins, F. Cunha, I. Santos, N. Castro, R. P. Andrade, E. Monteiro

**Affiliations:** Centro Hospitalar Tondela-Viseu , Viseu, Portugal

## Abstract

**Introduction:**

Psychotherapy serves as the foundation of care for individuals with borderline personality disorder (BPD), with pharmacotherapy being regarded as a supplementary measure to be considered when necessary. In clinical practice, however, most of BPD patients receive medication.

A major problem in the treatment of BPD is the lack of compliance derived from the pathological impulsivity of BPD patients. The use of long-acting antipsychotics (LAI) may be an option.

**Objectives:**

This work aims to address the use of long-acting injectables in borderline personality disorder.

**Methods:**

Non-systematic review of literature using the PubMed ® database, based on terms “Borderline Personality Disorder” and “Long-acting antipsychotics”. Only six articles were found.

**Results:**

Several studies have shown promising results in the treatment of Borderline Personality Disorder (BPD) with long-acting injectable (LAI) antipsychotics. A six-month study using IM risperidone demonstrated significant improvement, while LAI Aripiprazole also exhibited positive outcomes in individuals with BPD and Substance Abuse. Additionally, Palomares et al. (2015) found that palmitate paliperidone LAI reduced impulsive-disruptive behaviors and enhanced overall functioning in BPD patients. Carmona et al. (2021) compared oral and LAI antipsychotics and concluded that LAIs may have a role to play in the management of BPD.

**Conclusions:**

Treatment with LAIs may play an important role in clinical and functional improvement in BPD patients.

**Disclosure of Interest:**

None Declared

